# Content validity of the Patient-Reported Outcomes Measurement Information System Sleep Disturbance and Sleep Related Impairment item banks in adolescents

**DOI:** 10.1186/s12955-016-0496-5

**Published:** 2016-06-18

**Authors:** Jojanneke A. M. C. van Kooten, Caroline B. Terwee, Gertjan J. L. Kaspers, Raphaёle R. L. van Litsenburg

**Affiliations:** Department of Pediatric oncology - hematology, VU University Medical Centre Amsterdam, Amsterdam, The Netherlands; Department of Epidemiology and Biostatistics, VU University Amsterdam, Amsterdam, The Netherlands

**Keywords:** Sleep, Adolescent, Child, PROMIS, Validation

## Abstract

**Background:**

Sleep problems are common in adolescents and can have a negative impact on daily functioning and quality of life; therefore recognition of sleep problems is important. The PROMIS (Patient-Reported Outcomes Information System) Sleep Disturbance (SD) and Sleep Related Impairment (SRI) items banks are internationally used, well-validated instruments developed for and tested in adults. This study evaluates the content validity of the self- and proxy versions of the PROMIS-SD and the PROMIS-SRI in adolescents.

**Methods:**

Experts (*n* = 6), adolescents (*n* = 24, 12–18 years) and their parents (*n* = 7) commented on the relevance and comprehensibility of the item banks.

**Results:**

Experts considered all items relevant, only a few items were found irrelevant by adolescents and parents. The majority of items were comprehensible. The ability of parents to report on their adolescent’s sleep was limited.

**Conclusion:**

The PROMIS-SD and PROMIS-SRI have adequate content validity in adolescents. Considering their psychometric robustness and the possibility of Computerized Adaptive Testing, which is efficient as well as patient-friendly, these item banks could prove very useful in the evaluation of adolescent sleep. The validity of the proxy scales, however, is limited considering the difficulties reported by the parents. Further psychometric evaluation of these scales in adolescents is required.

## Background

The transition from childhood to adulthood leads to a change in sleep behavior typical for the adolescent population, such as later bedtimes, shorter sleep duration and sleeping in on weekends [1–3]. Sleep problems frequently occur in this population and can have a large impact on daily functioning [4, 5]. In a high school cohort, 21 % of adolescents self-reported a sleep problem [6]. Chung et al. found a prevalence of 5.6 % for difficulty falling asleep, 7.2 % for waking during night and 10.4 % for waking too early. Weekend delays in bed time and rise time were 64 and 195 min, respectively [7]. In the 2014 National Sleep Foundation Poll parents estimated a total sleep time of less than 7 h in 56 % in 15–17 year old teenagers. Sleep quality was considered lower in adolescents than in younger children. Less regularity in bed time and wake time was found in older adolescents [8]. O’Brien et al. found that longer weekend delays and more reported sleep problems were associated with a higher level of risk taking behavior and lower academic performance [9]. Inadequate sleep has a negative effect on overall performance in school and working memory performance [8, 10]. It also leads to more daytime sleepiness, depressive mood and sleep/wake behavior problems and has a major impact on mood, behavior and quality of life [8, 11]. The presence of a television in the bedroom, the use of technologies, a lack of consistent parental sleep rules, social demands and academic pressure have been associated with poorer sleep [2, 8, 12–15]. Sleep problems are also common in children suffering from chronic illnesses and are known to negatively influence quality of life [16–18]. Therefore recognition of sleep problems and their consequences in healthy and sick adolescents at an early stage is important.

Aspects of sleep quality, quantity and preferences can be measured objectively with electroencephalography, polysomnography and actigraphy; or subjectively through questionnaires. Objective and subjective measurements are often complimentary, as questionnaires are less reliable when it comes to assessing sleeping times and time awake during the night, but are suitable to capture perceived aspects of sleep such as fatigue, daytime sleepiness, nightmares, fear (e.g. of sleeping alone) or bedtime resistance [19, 20]. Pediatric sleep questionnaires should preferably be available in self and (parent) proxy formats, as sick children and adolescents are not always capable of responding to questionnaires. In questionnaire-based studies self-reports and proxy-reports often show different results, especially on more subjective domains [6, 21]. Still, it can be important to gather proxy assessments as this can contribute to the understanding of sleep problems.

Questionnaires with good psychometric properties are needed to reliably assess subjective quality of sleep and the consequences of inadequate sleep. The Patient-Reported Outcomes Measurement Information System (PROMIS) sleep disturbance (SD) item bank and sleep related impairment (SRI) item bank have been developed through a systematic process of literature reviews, content-expert review, qualitative research, pilot testing, and psychometric testing. They have shown good validity in adults but have not yet been validated in adolescents [22].

Considering the robustness of the PROMIS-SD and PROMIS-SRI items banks and the increasing international implementation [23–25], establishing the usefulness of these item banks in adolescents may provide pediatric healthcare workers with a solid sleep assessment tool for this age group. Therefore, this study reports on the content validity of the PROMIS-SD and the PROMIS-SRI in adolescents. Additionally, since (parent) proxy versions can add valuable information, proxy versions of the PROMIS-SD and PROMIS-SRI were developed and the content validity was assessed.

## Methods

### PROMIS item banks

PROMIS was developed by a National Institutes of Health (NIH) - funded consortium. This consortium aims to build item pools and develop core questionnaires that measure key health-outcome domains manifested in a variety of chronic diseases [22]. The item banks are developed through a systematic process of literature reviews, content-expert review, qualitative research, pilot testing, and psychometric testing. The PROMIS-SD and the PROMIS-SRI were developed to evaluate sleep. The PROMIS-SD item bank consists of 27 items and assesses perceptions of sleep quality, sleep depth, and restoration associated with sleep; perceived difficulties and concerns with getting to sleep or staying asleep; and perceptions of the adequacy of and satisfaction with sleep. The PROMIS-SD does not include symptoms of specific sleep disorders, nor does it provide subjective estimates of sleep quantities (e.g., the total amount of sleep, time to fall asleep, or amount of wakefulness during sleep) [26]. The PROMIS-SRI item bank contains 16 items and assesses perceptions of alertness, sleepiness, and tiredness during usual waking hours, and the perceived functional impairments during wakefulness associated with sleep problems or impaired alertness. The PROMIS-SRI measures the level of waking alertness, sleepiness, and function within the context of overall sleep-wake function, but does not directly assess cognitive, affective, or performance impairments [26]. The items can be administered in a questionnaire with fixed items or, more efficiently, through Computerized Adaptive Testing (CAT). With CAT the computer selects questions from an item bank, based on the answers to previous questions [27]. Both item banks have good face validity and construct validity [22]. The item banks were translated to Dutch-Flemish using standardized methodology and approved by the PROMIS Statistical Center. The translation included four forward translations, two back translations, independent reviews and pre-testing in 70 adults from the Netherlands and Flanders [24]. For the parent-proxy versions of the PROMIS-SD and PROMIS-SRI, the subject in all items was changed from ‘I’ to ‘my child’. The construct measured by the questions was not altered.

### Participants and procedure

Adolescents aged 12–18 years and their parents were recruited through secondary schools and the social networks of the authors and their colleagues. The exclusion criteria were insufficient understanding of the Dutch written language and a lack of informed consent. Twenty-four adolescents participated (12–13 years *n* = 7; 14 years *n* = 2; 15 years *n* = 3; 16 years *n* = 1, 17–18 years *n* = 11). Fifteen participants were recruited from secondary schools and nine from the authors’ social network. The cohort represents all three major Dutch secondary educational levels from different areas of the Netherlands. Nineteen parent-proxy questionnaires were distributed of which nine were returned. Seven parent-proxy reports could be used for the final analysis (37 %) since one was returned empty and one was discarded due to unclear answers.

The experts on sleep consisted of five Dutch clinicians and researchers, including one of the authors (RLI). A sixth expert on clinimetrics (author CTW) was also included in the expert-panel.

The adolescents and their parents received paper versions of the PROMIS-SD and the PROMIS-SRI. The participants were instructed to reflect on the relevance and comprehensibility of the item banks, see section on content validity. To prevent the participants filling out the questionnaire instead of evaluating it, response options were not provided.

The questionnaires were distributed to the adolescents in the classroom. One of the researchers gave the above described instructions and then stayed throughout the time the adolescents needed to evaluate the questionnaire to oversee the process and provide clarity where needed. The adolescents received a proxy version and stamped return envelope for their parents. Participants from the authors’ social network received the questionnaires by mail together with an instruction letter. The experts were contacted by email.

The study was approved of by the medical ethical review board of our institution.

### Content validity

Requirements for good content validity were adopted from the COnsensus-based Standards for the selection of health status Measurement INstruments (COSMIN) checklist. The COSMIN checklist was developed to evaluate the methodological quality of studies on measurement properties [28, 29]. The following criteria were evaluated: 1) All items should refer to relevant aspects of the construct to be measured; 2) All items should be relevant for the study population; 3) All items should be relevant for the purpose of the measurement instrument; 4) All items together should comprehensively reflect the construct to be measured.

To assess whether the PROMIS-SD and the PROMIS-SRI met these criteria, the adolescents, their parents and the experts were invited to comment on the relevance of the items for measuring subjective quality of sleep and consequences of inadequate sleep in adolescents. Comprehensibility was determined by asking the adolescents for each item whether they were clear and unambiguous. Finally, the parents were asked to indicate for each item whether they had enough insight into their adolescent’s sleep to adequately respond.

### Analysis

The participants’ input on the relevance and comprehensibility of the PROMIS-SD and the PROMIS-SRI was evaluated as follows.

Relevance of the items: All items marked as irrelevant for adolescent sleep by the experts were considered, since the sleep experts are best able to oversee the field of sleep issues in this population. For the responses from the adolescents and their parents, only items marked as irrelevant by at least 30 % of adolescents or their parents were selected. This threshold was chosen to prevent personal sleep issues from having too large an impact on the perceived relevance of items.

In order to maximize the comprehensibility of the questionnaires to the adolescents, a threshold of 90 % was employed; items were considered to be comprehensible if indicated as such by over 90 % of adolescents. To determine whether parents had enough insight into their child’s sleep to adequately answer each item, a threshold of 30 % was employed in order to prevent personal circumstances from having too large an impact on the results. Items were considered too difficult for parents to answer on behalf of the adolescents, if indicated so by over 30 % of the parents.

## Results

### Relevance

According to the experts, none of the items of the PROMIS questionnaires were irrelevant. Seven items from the PROMIS-SD were considered irrelevant by over 30 % of adolescents. Two items from the proxy-version of the PROMIS-SD and one from the PROMIS-SRI were considered irrelevant by over 30 % the parents. See Table 1.

**Table 1:** Items considered irrelevant by respondents

Respondents	PROMIS-SD and PROMIS-SRI items
Adolescents^a^	- My sleep was restful^c^
	- My sleep was refreshing^c^
	- I felt worried at bedtime^c^
	- I was afraid I would not get back to sleep after waking up^c^
	- My sleep quality was…^c^
Parents^a^	- My child’s sleep was refreshing^d^
	- My child’s sleep quality was…^d^
	- When my child woke up, he/she felt ready to start the day^e^
Experts^b^	-

^a^Items were considered irrelevant if reported by ≥30 % of the adolescents or parents; ^b^Items were considered irrelevant if reported by any of the experts; ^c^Items originating from the PROMIS Sleep Disturbance item bank; ^d^Proxy version of items from the PROMIS Sleep Disturbance item bank; ^e^Proxy version of items from the PROMIS Sleep-Related Impairment item bank

### Comprehensibility

Four items from the PROMIS-SD scale were marked by over 90 % of the adolescents as being too difficult: “My sleep quality was…”; “I got enough sleep”; “My sleep was refreshing” and “I had trouble staying asleep”. None of the items from the PROMIS-SRI were considered too hard to understand.

### Parent proxy responses

Parents often indicated insufficient insight into their adolescent’s quality of sleep and consequences of inadequate sleep to adequately answer the questions, see Table 2. Only two of the 27 (7 %) PROMIS-SD items could be answered by all parents and 24 items (89 %) reached the threshold of 30 % of parents not being able to answer. Parents reported fewer difficulties with the PROMIS-SRI items.

**Table 2:** Ability of parents to respond to the items

Questionnaire	Percentage of items answered by all parents	Percentage of items ≥30 % parents could not answer
PROMIS Sleep Disturbance (SD)	7	89
PROMIS Sleep Related Impairment (SRI)	31	38

## Discussion

Adolescents are mentally and physically transitioning from child to adult. This makes adolescents a very distinct population, also with regards to sleep behavior. Sleep problems frequently occur in this population and are known to have an extensive impact on daily functioning [8–11, 18]. Therefore, early recognition is important. The PROMIS-SD and PROMIS-SRI item banks are well validated and internationally used instruments. They were developed for and tested in adults [22–25] but could, considering their robustness, be very useful in adolescents as well. This study reports on the content validity of the PROMIS-SD and the PROMIS-SRI in adolescents.

For the relevance of the items to measure the construct of interest, the opinion of the experts was considered most important. They judged all items on the PROMIS-SD and the PROMIS-SRI to be relevant for adolescent quality of sleep and consequences of inadequate sleep. Adolescents reported seven PROMIS-SD items to be irrelevant of which two were also considered difficult. This may have influenced their assessment of relevance. No items on the PROMIS-SRI were irrelevant according to the adolescents. Parent-proxies found only few PROMIS-SD and PROMIS-SRI items irrelevant. Considering the experts’ opinion, the low number of parent-proxy respondents and the international purpose, construct and validation of the PROMIS scales, no alterations to the PROMIS-SD and PROMIS-SRI were made at this stage. The adolescents reported little difficulties with the comprehensibility of the items. Four PROMIS-SD items were regarded as too difficult. Since we did not provide the respondents with the answers options, the item ‘My sleep quality was…’ may have seemed an open-ended question and therefore difficult to understand. The question ‘I got enough sleep’ is very subjective and may therefore have been found less comprehensible. To preserve the construct of the PROMIS questionnaire the items were not removed. For the remaining two questions (‘My sleep was refreshing’ and ‘I had trouble staying asleep’) the Dutch translation may have been too complicated for adolescents. Alternatives items (‘My sleep gave me new energy’ and ‘I had trouble staying asleep at night’, respectively) were formulated and will be compared to the original items in a subsequent validation study.

Parents were asked to indicate whether they had enough knowledge on their child’s sleep to adequately respond to the items. Most problems were reported on the PROMIS-SD items compared to the PROMIS-SRI. This could be related to the more subjective nature of the items regarding sleep disturbances [6, 21], although the results could also have been influenced by the small number of parent-proxies.

The limitations of this study include a limited number of participants, especially a small group of parent-respondents. Adolescents with known sleep problems, visiting for example a sleep clinic, were not represented in the study group. However, small sample sizes are often considered sufficient in content validity studies [30, 31]. Also, the sample does represent a group of adolescents of different ages and education levels from different parts of the Netherlands that can be considered representative for a general adolescent population. A threshold of 30 % determined the difficulties parents experienced to report on their adolescent’s sleep. This threshold was, however, chosen by the authors as guidelines do not exist. Response options were not given to prevent the participants from filling out the questionnaire instead of evaluating it. This may, however, have impaired the comprehension of some of the items. Finally, it is important to note that the PROMIS item banks were not designed to assess specific characteristics of adolescent sleep, such as sleep time irregularities, screen time and social/academic pressures. A separate instrument would be needed to capture these aspects.

## Conclusions

The PROMIS-SD and PROMIS-SRI have adequate content validity in adolescents. Considering their psychometric robustness and the possibility of CAT administration, these item banks could prove very useful in the evaluation of adolescent sleep. The validity of the proxy scales is limited considering the difficulties reported by the parents. Further psychometric evaluation of these scales in adolescents is required.

## Abbreviations

CAT, Computerized Adaptive Testing; COSMIN, COnsensus-based Standards for the selection of health status Measurement INstruments; NIH, National Institutes of Health; PROMIS, Patient-Reported Outcomes Measurement Information System; SD, Sleep Disturbance; SRI, Sleep Related Impairment
